# Novel Apoptotic Mediators Identified by Conservation of Vertebrate Caspase Targets

**DOI:** 10.3390/biom10040612

**Published:** 2020-04-15

**Authors:** Nina Gubina, Dominique Leboeuf, Konstantin Piatkov, Maxim Pyatkov

**Affiliations:** 1 Institute of Theoretical and Experimental Biophysics, Russian Academy of Sciences, Pushchino, 142290 Moscow, Russia; 2 Center fof Life Sciences, Skolkovo Institute of Science and Technology, 121205 Moscow, Russia; Dominique.Leboeuf@skoltech.ru; 3 Center for Neurobiology and Brain Restoration, Skolkovo Institute of Science and Technology, 121205 Moscow, Russia; K.Piatkov@skoltech.ru; 4 Institute of Mathematical Problems of Biology, Keldysh Institute of Applied Mathematics, Russian Academy of Sciences, Pushchino, 142290 Moscow, Russia

**Keywords:** caspases, apoptosis, cleavage site, N-degron pathway, conservation, evolution, regulation

## Abstract

Caspases are proteases conserved throughout Metazoans and responsible for initiating and executing the apoptotic program. Currently, there are over 1800 known apoptotic caspase substrates, many of them known regulators of cell proliferation and death, which makes them attractive therapeutic targets. However, most caspase substrates are by-standers, and identifying novel apoptotic mediators amongst all caspase substrates remains an unmet need. Here, we conducted an in silico search for significant apoptotic caspase targets across different species within the Vertebrata subphylum, using different criteria of conservation combined with structural features of cleavage sites. We observed that P1 aspartate is highly conserved while the cleavage sites are extensively variable and found that cleavage sites are located primarily in coiled regions composed of hydrophilic amino acids. Using the combination of these criteria, we determined the final list of the 107 most relevant caspase substrates including 30 novel targets previously unknown for their role in apoptosis and cancer. These newly identified substrates can be potential regulators of apoptosis and candidates for anti-tumor therapy.

## 1. Introduction

Caspases are cysteine proteases that mediate a vast array of cellular processes and are best known for their prominent role in initiation of inflammation and executing programmed cell death. Unlike other proteolytic enzymes, caspases are extremely specific and cut only a selected set of proteins [1]. Caspase cleavage can result in protein degradation, activation or a change in the cellular localization of the protein, depending on the newly generated N-terminal signal [2]. Mutations leading to insufficient or inefficient caspase activation are associated with compromised cell death and carcinogenesis [3,4], whereas over-activation of caspase-dependent proteolysis aggravates cell death and leads to atrophy, in particular, to neurodegeneration [5]. Accordingly, manipulation of caspase activity was explored in cancer therapy to increase tumor cell death or, conversely, to prevent apoptosis in diseases such as chronic hepatitis C virus (HCV) infection or Alzheimer’s disease [6]. Therefore, a rigorous study of caspase substrates will serve to identify appropriate drug targets, especially if caspases and upstream factors of apoptosis are not available for direct manipulation [7].

Currently, the number of known human proteins cleaved by apoptotic caspases is over 1800 [7]. Some caspase substrates are key players in the propagation of cell death contributing to observable morphological changes and taking part in positive feedback loops that increase the efficiency or robustness of the process [2]. Other caspase substrates, however, are simply by-standers of the apoptotic process [8]. It is technically difficult to separate cleavage events relevant for apoptosis from by-stander cuts using standard wetlab technologies, such as cleavage site mutants [1,9]; thus, the complete critical subset of proapoptotic caspase targets has yet to be determined. An in silico evolutionary approach could shed light on this problem and provide the basis for studying caspase substrates in vitro and in vivo. 

Apoptosis is a highly conserved process; therefore, conservation of an apoptotic caspase target suggests functional significance, whereas a non-conserved target is more likely to be a by-stander. Additionally, the nature of the newly formed N-terminus could also be an indication of the importance of the substrate in the proapoptotic program [10]. Thus, examining the conservation patterns of caspase cleavage targets across different taxa would define the set of the most significant caspase targets and address the functional importance of cleavages on a broad scale [2,11]. 

Attempts to estimate conservation of caspase targets were made once in vitro [12] and once in silico [13]. In the first attempt, the authors initiated apoptosis in human, mouse, fly, and worm cell lines and identified the resultant cleavage fragments [12]. However, the intersection between all four sets of fragments was small, partly because the species were from too distant taxa. In the second study, the authors took the whole set of human proteins known as caspase targets, found orthologs, and estimated the conservation of aspartate and glutamate cuts within the whole Metazoan taxon [13]. They found that both P1 aspartate and glutamate cut sites are generally stable during evolution, but they did not study conservation at the level of separate caspase substrates.

In the present study, we conducted an in silico search for significant apoptotic caspase targets using different criteria of conservation combined with structural features of cleavage sites. We used 60-amino acid sequences surrounding human cleavage sites as a query, on a large number of species but within the narrower evolutionary scale of the Vertebrata subphylum. We observed a highly conserved nature for the P1 aspartate but extensive variability in the eight amino acid cleavage sites, as was reported previously [12,13], and found that apoptotic caspase cleavage sites are located primarily in coiled regions composed of hydrophilic amino acids. Moreover, we found that 20% of caspase substrates have a potential built-in N-degron that gets exposed upon cleavage by caspases, indicating that the substrate may be subject to fast degradation through the N-degron pathway [10]. This destabilization is an important regulator for some proapoptotic caspase substrates [14]. Using the combination of these criteria, we determined the final list of the 107 most relevant caspase substrates including 30 novel targets previously unknown for their role in apoptosis and cancer that can be potential regulators of apoptosis and candidates for anti-tumor treatment.

## 2. Materials and Methods

All data analysis, statistical treatment, and visualization were completed using R free software (version 3.6.2) (R Foundation for Statistical Computing, Vienna, Austria), package “Tidyverse” [15]. Scripts are available online at Github. URL https://github.com/mpyatkov/caspases.

### 2.1. Gathering Human Apoptotic Caspases Cleavage Sites

Human apoptotic caspase cleavage sites were collected from existing databases: MEROPS [16], CutDB [17], Degrabase [18], CaspDB [19], and from the literature. Only experimentally approved sites obtained after direct activation of apoptosis by any caspase were considered; predicted targets and results of machine learning were excluded. Although caspases can cleave after glutamate [13], only P1 D-cut sites were kept for the following analysis, because the functional significance of P1 E-cut sites is still unclear. Obsolete Uniprot Ids, duplicates, and readthroughs were filtered out. Pseudogenes were kept if they had Uniprot evidence at the protein level. In the cases of several gene names with the same Uniprot ID, only one gene name was used in the subsequent data analysis (Table A1). The results are summarized in Table S1.

### 2.2. Search for the Vertebrate Orthologs of Human Apoptotic Caspase Cleavage Sites 

**Figure 1:**
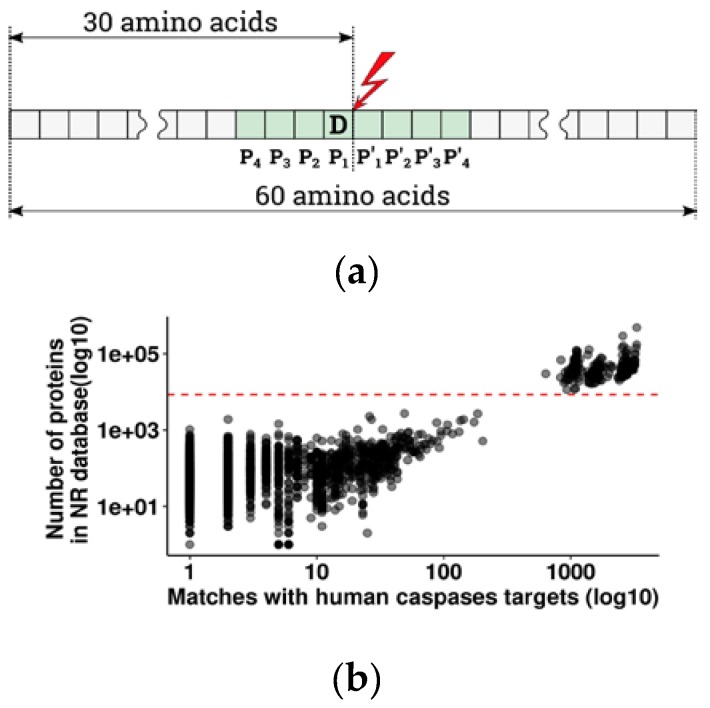
Selecting orthologous targets and representative proteomes. (**a**) Scheme of the 60 amino acid query sequences for the pBLAST search to identify human apoptotic caspase cleavage site orthologs. P4–P4′: positions of amino acids within the cleavage site. D: key aspartate in the P1 position. Red lightning: scissile bond between P1 and P1′ amino acids. Green elements: caspase cleavage site. (**b**) Distribution of species by number of sequenced proteins in a non-redundant database and number of matches with human caspase cleavage sites. Each point corresponds to one species. The group above the red dashed line embraces species with well characterized proteomes. The red dashed line represents the threshold for the number of annotated proteins used to separate species with a well characterized proteome (log10(total N of proteins) > 3.9, or total N of proteins > 8000).

A search for the orthologs of human apoptotic caspase cleavage sites was done using pBLAST [20], with default parameters and an e-value cut-off of 1 × 10^−16^ [13,21], and on the vertebrate subset of a non-redundant (NR) protein database. Input sequences for pBLAST, including human octamer cleavage sites +/- 26 surrounding amino acids for a total of 60 amino acids (Figure 1a), were retrieved from the Uniprot database [22] using an R script [23]. In similar studies [13,21], the authors used whole proteins as a query sequence to search for orthologs. We used partial sequences because they help to locate cleavage sites in orthologs more precisely while being representative for the search for structure similarity.

For each human query sequence, pBLAST found a batch of orthologs within a single species that are more likely duplicates from different experiments. We kept only one hit sequence per species with the best pBLAST e-value.

### 2.3. Selecting Species with a Well-Represented Proteome

Selection of the threshold for proteome representation was based on the distribution of species by total number of proteins in the vertebrate subset of a non-redundant database and the number of matches with 3363 human caspase targets (Figure 1b); 328 species with a well-represented proteome (N proteins per species > 8000) were selected for the following analyses.

### 2.4. Localization of Caspase Cleavage Site and P1 Aspartate in Orthologs

Orthologous cleavage sites and P1 amino acids were located using Hamming distance estimation [24] between human and orthologous 60-amino acids sequences with P1 aspartate as an anchor. This approach is algorithmically simpler and faster than using any type of alignment. For eight-amino acid sequences, Hamming distances range from 0 to 8, 0 meaning that there are no differing amino acids and that the sequences are identical, 8 meaning that all eight amino acids are different.

### 2.5. Obtaining Lineage Information

Lineages were originally retrieved from NCBI using TaxonKit [25] and then supplemented with information from the EMBL–EBI Taxonomy Service [26]. 

### 2.6. Determination of the Stabilizing/Destabilizing Effect of the P1′ Amino Acid

The P1′ amino acid becomes the nascent N-end after caspase cleavage and thus becomes a fate determinant of the C-terminal fragment. Denominations of stabilizing/destabilizing effects for P1′ amino acids were taken from [27]. 

### 2.7. Prediction and Analysis of the Secondary Structure

Secondary structure prediction was performed using the web-service MUFOLD, the best program by quality and speed [28]. As a query, we used the same human 60-amino acid sequences that were used to search for orthologs. The results of prediction were described in Q3 accuracy terms: helix (H), strand (E), and coil (C) [28]. Sequence logos were plotted using the free online software WebLogo [29].

### 2.8. Estimation of Hydrophobicity Indices

Hydrophobicity indices are defined as the free energy required to transfer amino acid side-chains from cyclohexane to water and are expressed as kilo-calories per mole. The indices for each of the 20 amino acids, in a distribution from non-polar to polar at pH = 7, were taken from Radzicka & Wolfenden [30].

### 2.9. Pathway Analysis

The final list of 99 proteins containing 107 conserved apoptotic caspase targets was submitted to the DAVID free online software [31]. Pathway enrichment was performed for two sets of annotation terms: Gene Ontology [32] and Uniprot [22], with post-hoc adjustment by Benjamini–Hochberg correction. Adjusted p-values less than 0.05 were taken as significance threshold for enrichment.

### 2.10. Statistics

Evaluation of statistical significance for boxplots was performed using analysis of variance (ANOVA) followed by Tukey’s post-hoc test. Asterisks indicate significance levels. * *p* < 0.05, ** *p* < 0.01, *** *p* < 0.001. The correlation analysis was performed using the Kendall rank correlation coefficient. A *p*-value less than 0.001 was taken as a significance threshold. Comparison of dendrograms obtained after hierarchical clustering of cleavage sites and of orthologous 60 amino acid sequences was performed using Baker’s Gamma correlation coefficient (Goodman–Kruskal–gamma index [33]). 

## 3. Results and Discussion

The aim of this study was to identify previously unknown apoptotic caspase substrates with a high functional significance in the apoptotic program using different parameters of conservation of the substrate as an indicator of importance. Each selected criterion will be explained in more detail below. 

We collected all experimentally derived human apoptotic caspase targets—3363 cleavage sites from 2040 proteins (Table S1)—and used them as a query to find orthologs in vertebrates (the details of selection are described in the Methods and Figure 1a). Similar approaches were previously used by Pearlman et al. [21] to trace the evolution of phosphorylation sites and by Seaman et al. [13] to estimate the significance and evolutionary conservation of cleavage sites with glutamate in the P1 position, in Metazoans. From the overall 44 885 vertebrate species present in the non-redundant protein database with at least one known protein, either sequenced or predicted from DNA and RNAseq data, 2875 species had at least one caspase cleavage target orthologous to human (pBLAST e-value < 1 × 10^−16^). Since the number of orthologous targets depends on the number of known proteins for a given species (Figure 1b), we chose to exclude species with poorly and unevenly represented proteomes in order to avoid false negative results. For further analysis, we selected 328 species with a well-represented proteome (more than 8000 annotated proteins per species) (Figure 1b). Due to misrepresentation, four large taxa are totally absent from that group: Myxinidae (hagfishes), Petromyzontidae (lampreys), Ceratodontimorpha (lungfishes), and Cladistia (Polypterus and reedfish). The refined results are listed in Table S2 (with a legend in Table A2) and represent the basis for the following search of conserved caspase substrates.

### 3.1. Vertebrate Caspase Targets are Highly Conserved at the Level of P1 Aspartate.

Caspases cleave proteins at the scissile bond between P1-P1′ of an octamer amino acid sequence P4-P3-P2-P1-P1′-P2′-P3′-P4′, with a strict requirement for aspartate in position P1 (Figure 1a) [34]. Indeed, analysis of amino acid distribution in the P1 position in vertebrates has shown that in 92% of orthologous targets, the P1 position is occupied by an aspartate residue (Table 1, Table S2). In most cases, substitution of the D in position P1 prevents or slows down the cleavage [13,35,36], and such proteins can be considered as non-conserved within the apoptotic pathway. Therefore, we decided to take the presence of aspartate in the P1 position as a prerequisite for cleavage conservation and as a threshold for further selection of the caspase targets most important for apoptosis.

Interestingly, around 5% of vertebrate cleavage site orthologs have glutamate in the P1 position instead of aspartate (Table 1). Previous in vitro reports have shown that caspases-3, -6, and -7 are able to accommodate P1 glutamate in the active site and cleave substrates with P1 E cut sites with the same affinity but with a twofold slower rate than substrates with aspartate in the same position [13,37]. The same study by the Wells group demonstrated that substitution of P1 glutamate by aspartate actually results in a higher rate of protein cleavage by caspases. The shared ability of caspases-3, -6, and -7 to cleave substrates after glutamate could be explained by their very similar structure, which originates from a single ancestral effector caspase gene [38]. Both P1 aspartate and glutamate appear to be conserved throughout Metazoans in general [13]; however, a taxon-oriented study showed that mammals have a lower incidence of P1 E-cut sites (3%) compared to birds (7%) and ray-finned fish (9%) (Table S3, Figure S1). Further, although P1 E cut sites exist in all taxa, the physiological presence and role of these substrates in vivo are unclear. We suggest that a transition from glutamate to aspartate in the P1 position during the evolution of Vertebrata is most likely an adaptive feature intended to make apoptosis more aggressive, which would ensure a faster elimination of damaged cells.

### 3.2. Low Level of Conservation of Apoptotic Caspase Cleavage Sites in Vertebrates

The numerical estimates of cleavage site similarity were calculated using the Hamming distance metric (HD) [24]. This metric is position-dependent and determines the difference between elements of two sequences for each position as True and False, or 0 and 1. For eight-amino acid sequences, the HD ranges from 0 to 8, where 0 corresponds to an absolute identity of sequences and 8 to no amino acid matches. 

Unlike for the key aspartate, the whole cleavage site sequences are not well preserved in vertebrates: although 92% of all found cleavage sites in vertebrates have aspartate in the P1 position, only 57% of orthologs have an ideal caspase cleavage site where all eight amino acids match their human counterparts, and 18% differ in one amino acid (Table 2). This finding correlates with earlier observations on smaller datasets showing that conservation of the entire cleavage site is weaker than conservation measured by the retention of aspartate in position P1 or by the primary structure [12,13,39].

The non-conserved nature of cleavage site sequences in caspase targets allowed us to recapitulate the phylogenetic relationship among vertebrates at the level of classes using the orthologous cleavage sites with almost the same accuracy as using the 60-amino acid orthologous sequences found by pBLAST (Baker’s Gamma correlation coefficient [33] was 0.913, Figure S2). However, this method of evolutionary analysis did not have enough resolution to track the phylogenetic tree back to orders, families, and genera (Figures S3 and S4). This may be explained by the observation that precise determination of phylogenetic relationship usually requires using much longer sequences, such as whole genes or whole genomes [40]. Nevertheless, the variability observed in the eight-amino acid sequences was enough to separate the species into classes. 

In summary, we calculated the median HD for each caspase target present in Table S1 among all orthologous D-cuts (Table S4) and used the resultant numerical range as a conservation parameter for further selection of caspase targets that are most important for apoptosis.

### 3.3. Human Caspase Cleavage Sites are Located Preferentially in Coiled Regions

**Figure 2:**
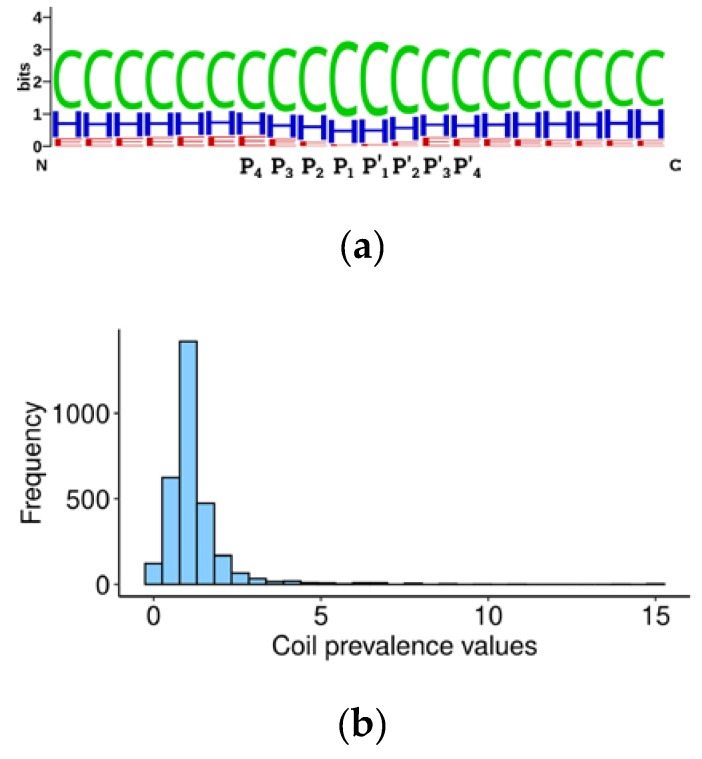
Secondary structure of the human caspase cleavage site surroundings. (**a**) Weblogo representation of the frequency of secondary structure elements surrounding the cleavage site (positions P4-P4′). One stack of letters corresponds to one amino acid. Secondary structure elements are abbreviated as follows: C: coils, H: helices, E: beta-strands. (**b**) Distribution of coil prevalence values of human caspase cleavage sites, calculated as described in the text.

Structure analysis of cleavage sites in both human [39] and *Escherichia coli* proteins [41] revealed that caspases cut proteins predominantly in disordered regions or coils, to a lower extent in α-helices and rarely in β-sheets. This happens because substrates can be cleaved only when in an extended conformation [42], and the loop regions are easier to unfold locally, compared to α-helices and β-sheets which often require global unfolding of the protein [43]. We calculated secondary structures for all human 60-amino acid sequences from Table S2 and characterized them using Q3 accuracy symbols: α-helix (H), β-sheet (E), and coil (C) [28]. The results are detailed in Table S5. The Weblogo alignment [29] of secondary structure elements around cleavage sites (20 amino acids) showed that 60% of elements are represented by coils, 30% by α-helices, and around 10% by β-sheets (Figure 2), in accordance with earlier observations [39,41,44] (Table S6).

We further developed the idea of caspase structural preferences and hypothesized that the substrate cleavage site should be more accessible for proteolysis if it is located not only in an unstructured region, but within the loop between two structured regions. Accordingly, we calculated the prevalence of coils in the central 20 amino acid sequences surrounding the P1 aspartate over marginal 20 amino acid sequences (Table S4) using the following formula:

(1) $$\mathrm{Prevalence}\;\mathrm{value}=\frac{2c}{l+r}$$

where ‘c’ is the percentage of coils in the central 20 amino acids, ‘l’ in the left, and ‘r’ in the right 20 amino acids. The obtained value should be proportional to the probability of the cleavage site location being in an unstructured loop between domains and, hypothetically, to the efficiency of cleavage. Consequently, this value should contribute to the overall significance of the substrate in programmed cell death. Curiously, the prevalence value for the two thirds of the human caspase targets is around 1, suggesting that there is mostly no difference between the percentage of coils in regions immediately surrounding the cleavage site and in more distant sequences (Figure 2b). A coil prevalence value higher than 1 will be tested later as a criterion to select caspase targets which are most relevant for apoptosis.

### 3.4. Most Vertebrate Caspase Cleavage Sites are Located within Hydrophilic Surroundings

Proteins in aqueous conditions, such as the cytosol, tend to have hydrophobic residues hidden within their structure and hydrophilic amino acids exposed to the surface [45]. This feature suggests that proteolysis will most likely happen within the hydrophilic portions of proteins, because these exposed parts would be more accessible for cleavage. Thereafter, hydrophilicity of cleavage sites facilitating caspase digestion of the protein would make the respective substrates more important for apoptosis.

**Figure 3:**
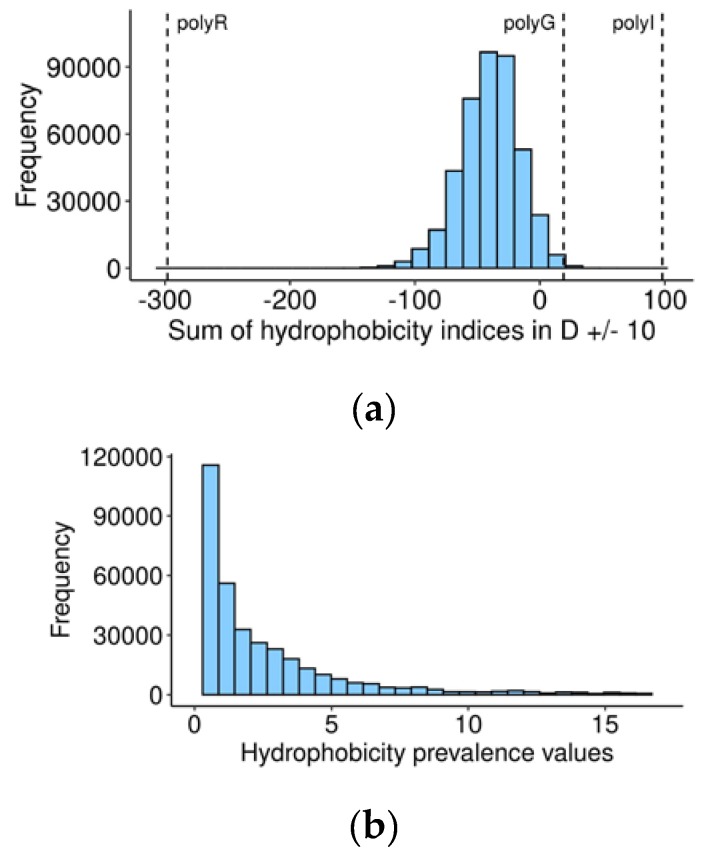
Hydrophobic properties of vertebrate cleavage sites. (**a**) Distribution of the sums of hydrophobicity indices (HI) for the 20 amino acids surrounding P1 aspartate in all caspase substrates. PolyR: polyarginine-20 (HI = −298.4), hydrophilic limit of the scale. PolyI: polyisoleucine-20 (HI = 98.4), hydrophobic limit of the scale. PolyG: polyglycine-20 used as a reference to designate a borderline between hydrophobic and hydrophilic peptides. It was chosen because its HI (0.94) is the closest to the median HI among all 20 amino acids (0.4). (**b**) Distribution of hydrophobicity prevalence values in all caspase substrates, calculated as described in the text.

Exploring this possibility, we calculated the sum of hydrophobicity indices (kilo-calories per mole for each of the 20 amino acids at a pH of 7) [30] for the central 20-amino acid sequences with P1 aspartate in the middle, for every human and orthologous 60-amino acid sequences (Table S2). On the suggested scale, a negative value represents hydrophilicity, while positive suggests hydrophobicity. Most of the vertebrate caspase cleavage sites are located in a hydrophilic environment, as expected (Figure 3a), and are likely exposed at the surface of the protein. Nevertheless, non-hydrophilic cleavage sites may relocate to the surface and become available to caspases under certain conditions, leading to a change in conformation, for example, oxidative stress and the unfolded protein response, which often precedes cell death [46,47]. 

Additionally, we found a negative correlation between the hydrophobicity of human cleavage sites and cleavage rates of the corresponding caspase targets in human cells [48], so that hydrophilic sites tend to be cut faster than hydrophobic sites (Figure S5, Kendall rank correlation coefficient = −0.233, *p*-value = 1.082173 × 10^−5^). As it was previously demonstrated that a shorter half-life is associated with a more potent regulatory outcome [49], the higher hydrophilicity of the cleavage site could contribute to the overall importance of the substrate for apoptosis by facilitating cleavage.

As in the case of secondary structures, we hypothesized that localization of the cleavage site in a hydrophilic loop between less hydrophilic segments would increase the chances of the substrate to be cut, and calculated for each vertebrate caspase target the hydrophobicity prevalence in the central 20 amino acids over averaged marginal fragments with the same formula used to calculate the prevalence of coils (1), where ‘c’ is the sum of hydrophobicity indices in the central 20 amino acids, ‘l’ in the left, and ‘r’ in the right 20 amino acids. Prior to calculating this value, we shifted the distribution of (l + r)/2 and c values to make them all positive. The magnitude of the shift was calculated using the following formula:

(2) $$\mathrm{Shift}=\left|min\left(min\frac{\vec{l+r}}{2},min\vec{c}\right)\right|+1$$

where we selected the minimum of (l + r)/2 and c values calculated for all vertebrate targets and added 1 to this minimum, to avoid possibly dividing the final formula by zero. The resultant shift value was added to each (l + r)/2 and c. Hydrophobicity prevalence values for all vertebrate orthologs of human caspase cleavage sites are presented in Table S2. In the resultant range, a value less than 1 indicates that the cleavage site area is more hydrophilic than flanking regions. Remarkably, the distribution of these values (Figure 3b) has a peak around 1, indicating that in most human caspase targets, the cleavage site does not differ in hydrophilicity from flanking regions. 

Further, we calculated the median hydrophobicity prevalence for each caspase target (Table S4). A median hydrophobicity prevalence < 1 will be used in further analysis as a criterion to separate the most important apoptotic caspase targets from the by-standers.

### 3.5. Twenty Percent of Vertebrate Apoptotic Caspase Targets Have an Exposed N-degron after Cleavage

Apoptosis is a tightly regulated process and is highly influenced by the creation and degradation of apoptotic mediators generated by activated caspases. One of the degradation mechanisms for the caspase substrates is proteolysis mediated by the N-degron pathway [14]. The N-degron pathway selectively degrades protein fragments based on the recognition of specific destabilizing amino acid residues at their N-termini [10]. It was shown that several established proapoptotic factors activated by caspase cleavage have destabilizing amino acids in their nascent N-ends, and their ability to propagate cell death is counteracted by the N-degron pathway [14]. Therefore, evolutionary conservation of destabilizing amino acid residues at the N-end of caspase cleavage products, corresponding to the P1′ position in the caspase cleavage site, may indicate a potential regulatory function for the protein fragment and can be used to separate caspase targets most important for cell death.

Destabilizing residues formed after protein cleavage are recognized by the Arg/N-degron pathway directly (R, K, H, L, F, Y, W, I) or after being arginylated by the ATE1 arginyl-transferase (D, E, C directly, N, Q after tertiary modifications) [10]. Small residues such as P and G, or residues M, V, S, A, and T, that become destabilizing only after posttranslational Nt-acetylation [50], are not recognized by the Arg/N-degron pathway and are considered stabilizing residues [10]. We closely examined the identity of the P1′ position in all studied cleavage sites and found that both in humans and in 328 non-human vertebrates, the distribution of amino acids in the P1′ position of substrates is the same: ~30% Gly, 24% Ser, 14% Ala, with 32% distributed among the other amino acids (Table 3). Similar results were obtained in a peptide library study [51] and in apoptotic Jurkat cells [39], where G, S, and A were the most common residues observed after cleavage by caspases-1, -3, -6, -7, and -8. These amino acids are stabilizing, and only 20 percent of caspase targets in 328 vertebrate species are destabilized after cleavage (Table 3). However, nearly 100% of these 20 percent have conservation of the destabilizing nature of the P1′ residue among 328 non-human vertebrates (Table S4, Figure S6a). 

Curiously, among three classes of vertebrates, mammals have a lower proportion of destabilizing P1′ residues compared to birds and lower vertebrates (Table S7, Figure S6b). Similar trends were observed for the percentage of P1 glutamate cut sites (Table S3, Figure S1). Both of these evolutionary trends tend to make the apoptotic process more aggressive in higher vertebrates, by switching from glutamate to aspartate in the P1 site, which increases the rate of caspase substrate cleavage, and by shifting from a P1′ destabilizing to stabilizing amino acid, preventing the fragment from being degraded by the N-degron pathway, making it persist more and function longer. These trends can be explained by the increased complexity and redundancy of mechanisms of apoptosis regulation in higher vertebrates, reducing the need to rely on a less specific regulators of apoptosis such as the N-degron pathway [52,53,54]

In further analysis, maintenance of the destabilizing nature of P1′ in more than 50% of vertebrate orthologs will be used as a criterion of target importance for apoptosis.

### 3.6. New Potential Apoptotic Regulators and Candidates for Cancer Therapy

The ultimate goal of this work was to identify the subset of caspase targets that are the most important in the apoptotic program, based on conservation throughout evolution. In order to reach this goal, we first selected highly similar orthologous sequences, which have aspartate in the P1 position, and then elaborated four separating parameters: cleavage site similarity, structural disordinance, hydrophobicity of the cleavage site area, and conservation of the destabilizing nature of the P1′ position.

We examined the predictive power of these parameters when applied to a reference set of caspase targets of which the newly generated C-terminal fragments possess known proapoptotic activity: RIPK1, TRAF1 [14], CASP-2, -3, -6, -7, -8, -9, PARP1, PARP2, and ICAD [55] (Table 4). Only four of the 24 reference targets had a conserved destabilizing effect of the P’ amino acid (Table 3) and because of the limited compliancy of the reference set substrates to this particular criteria, we opted not to impose it on future analysis. On the other hand, most of the reference caspase targets have well conserved cleavage sites in hydrophilic loops and in unstructured regions, which makes these three criteria predictive enough to separate the most important caspase substrates.

To refine the results, we added two more criteria. First, as the Vertebrata subphylum encompasses seven classes, the presence of a caspase target in every class was considered an additional measure of conservation. Second, since there are 328 species in the filtered pBLAST output, we included having more than 96% orthologs for a caspase target as another indicator of conservation. Applying all five thresholds to vertebrate orthologs of human caspase targets (Table S2), we generated a final list of 107 caspase targets in 99 proteins that are most conserved among vertebrates and therefore should be the most important in the apoptotic program (Table S8). 

The main limitation of this approach resides precisely in the last two criteria: strict adherence to conservation and presence of the substrate in all seven classes implies that we are filtering out substrates that have evolved later over time. For instance, any substrate appearing after Actinopterygii would be eliminated. However, further pair-wise analysis of caspase substrates could shed light on class-specific apoptotic pathways. 

**Figure 4:**
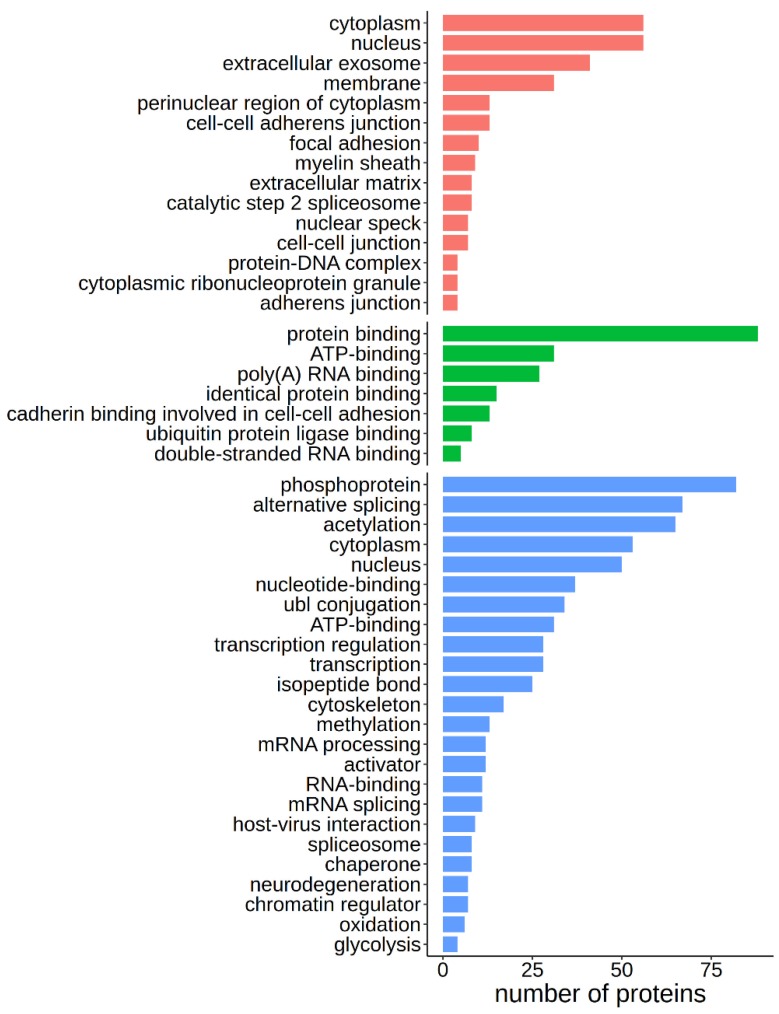
Pathway enrichment of the 107 highly conserved caspase targets. Pathway analysis using the DAVID free online software, with default parameters and an EASE threshold of <0.05 was followed by annotation using two databases: Gene Ontology [32] and Uniprot [22]. Proteins were classified in terms of Gene Ontology—Cellular Component (**red**), Gene Ontology—Molecular Function (**green**), and Uniprot Keywords (**blue**). The *p*-value for annotation selection was adjusted by Benjamini–Hochberg correction. Annotations with a corrected *p*-value < 0.05 were considered significant and were presented in the plots.

Approximately 51% of the caspase targets from the list (55/107) are known to be involved in apoptosis (Table S8). Some proteins, such as Caspase-7 (CASP7), Rho associated coiled-coil containing protein kinase 2 (ROCK2), and Protein Kinase N1 (PKN1), become activated upon caspase cleavage and proceed to propagate apoptosis [56,57,58]. Others, such as aryl hydrocarbon receptor nuclear translocator (ARNT) and ubiquitin specific peptidase 19 (USP19) are anti-apoptotic regulators, which are deactivated by caspase cleavage [59,60]. These results validated our method and confirmed our hypothesis that important proteins involved in apoptosis would be conserved throughout evolution and that conversely, evolutionary conserved caspase targets would be important in apoptosis. Further pathway analysis of the 107 conserved caspase targets using the DAVID free online software [31] with default parameters and an EASE threshold of 0.05%, followed by annotation using two databases, Gene Ontology [32] and Uniprot [22], allowed us to highlight the more prevalent pathways where these conserved caspase targets are involved. Significantly enriched terms include nucleus-related processes such as alternative splicing and RNA processing, phosphorylation, methylation, and acetylation, ATP binding, and protein interaction (Figure 4). The 107 caspase targets can be equally found in the cytoplasm or the nucleus, and enriched cellular locations include membranes and extracellular exosomes (Figure 4).

From the remaining targets (52/107), 22 were found to be involved in cancer, and an extensive literature search exposed 30 conserved caspase targets that have not been previously associated with apoptosis or cancer (Table 5). From those, many targets—ATXN2L, ESS2, QRICH1, RFC1, SF3A1, and more—are related to regulation of RNA transcription, splicing, and processing, RRFM2 and SRP54 are related to protein biogenesis, ENO1 and 3 are implicated in gluconeogenesis, NAA15 participates in angiogenesis, and MAP15 is involved in microtubule organisation (Table S8). Another interesting caspase target is CCT5. While the exact role of this protein in apoptosis has not been elucidated, recent studies indicated that this protein is significantly upregulated in several cancers [61,62], can serve as a tumor-associated antigen [63], and has been implicated in an anti-viral apoptotic response [64], suggesting an opportunity for the development of a new proapoptotic therapy.

## 4. Conclusions

In this study, we isolated and identified a subset of thirty likely regulators of apoptosis and potential drug targets in cancer. By studying the conservation of structural and functional features of apoptotic caspase targets and analysing them according to cleavage site similarity, structural disordinance, hydrophobicity of the cleavage site area, presence of a caspase target in every vertebrate class, and having more than 96% orthologs for a caspase target, we determined the list of caspase targets that are important in the apoptotic program. Of these, 30 meet all criteria but have undetermined roles in apoptosis so far. Future studies will uncover the part they play in the apoptotic program and will determine how these caspase substrates can become targets of therapy for cancer. 

## Figures and Tables

**Table 1:** Distribution of amino acids in the P1 position.

P1 Amino Acid	Number of Hits	% of Total
D	562,772	92.19
E	28,423	4.66
N	5402	0.88
G	4771	0.78
A	1836	0.30
S	1772	0.29
Gap (-)	1331	0.22
V	698	0.11
H	683	0.11
T	632	0.10
K	564	0.09
Q	535	0.09
Y	221	0.04
P	186	0.03
R	137	0.02
I	126	0.02
C	121	0.02
L	103	0.02
Unknown	45	0.01
F	33	0.01
M	33	0.01
D or N	2	0.00
W	1	0.00

**Table 2:** Distribution of orthologous caspase targets by cleavage site conservation and presence of aspartate in the P1 position.

P1 Amino Acid	Hamming Distance Estimates ^1^	Numberof Hits	% of Total
	0	346,816	56.82
	1	111,584	18.28
	2	57,620	9.44
Aspartate	3	27,071	4.43
	4	12,265	2.01
	5	5380	0.88
	6	1750	0.29
	7	286	0.05
	1	8894	1.46
	2	11,338	1.86
	3	10,704	1.75
Not	4	7816	1.28
aspartate	5	5074	0.83
	6	2650	0.43
	7	978	0.16
	8	201	0.03

^1^ Indication of conservation: 0 = sequences are identical; 8 = all eight amino acids are different.

**Table 3:** Distribution of amino acids in the P1′ position and the nature of the amino acid according to the Arg/N-degron pathway.

P1′ Amino Acid	Nature of aa ^1^	Number of Hits in Humans	% in Humans	Number of Hits in Vertebrates	% in Vertebrates
G	stab	1080	33	171,377	30
S	stab	862	26	131,638	23
A	stab	505	15	80,871	14
T	stab	92	3	20,011	4
L	destab	91	3	18,102	3
V	stab	83	3	17,729	3
F	destab	80	2	13,377	2
Y	destab	74	2	14,295	3
N	destab	65	2	18,225	3
D	destab	53	2	11,274	2
M	stab	50	2	8552	2
E	destab	46	1	9115	2
K	destab	41	1	7301	1
I	destab	40	1	7507	1
C	destab	36	1	6986	1
H	destab	31	1	6596	1
P	stab	28	1	9298	2
Q	destab	22	1	3929	1
R	destab	22	1	6320	1
W	destab	12	0	2754	0

^1^ stab: stabilizing after caspase cleavage, destab: destabilizing effect, according to the Arg/N-degron pathway.

**Table 4:** Validation of conservation criteria based on the set of reference caspase targets with proven proapoptotic activity ^1.^

	Criteria			
	Median Hamming Distance < 2	Coil Prevalence > 1	Hydrophobicity Prevalence < 1	P1′ Destabilizing in > 50% of Orthologs
Number of human targets selected by threshold	2726	1454	1457	554
Number of reference targets selected by threshold	19	17	16	4
Percentage of human targets selected by threshold	91.48	48.79	48.89	18.59
Percentage of reference targets selected by threshold	79.17	70.83	66.67	16.67

^1^ The set of reference targets included 24 cleavage sites within 11 proteins: CASP2, CASP3, CAPS6, CASP7, CASP8, CASP9, PARP1, PARP2, RIPK1, TRAF1, CAD (Table S1).

**Table 5:** Novel apoptotic mediators not previously related to cancer and apoptosis.

Human Gene Name	Human Protein Symbol	Human Uniprot ID	Human Cleavage Site	Nature of P1′ ^1^
actin, beta like 2(*ACTBL2*)	ACTBL	Q562R1	ELPDGQVI	Stab
actin gamma 1(*ACTG1*)	ACTG	P63261	ELPDGQVI	Stab
ataxin 2 like (*ATXN2L*)	ATX2L	Q8WWM7	LESDMSNG	Stab
chaperonin containing TCP1 subunit 5 (*CCT5*)	TCPE	P48643	VDKDGDVT	Stab
EH domain containing 4 (*EHD4*)	EHD4	Q9H223	CDCDGMLD	Stab
enolase 1 (*ENO1*)	ENOA	P06733	YGKDATNV	Stab
enolase 3 (*ENO3*)	ENOB	P13929	YGKDATNV	Stab
DiGeorge syndrome critical region gene 14 (*DGCR14*)	ESS2	Q96DF8	VGPDGKEL	Stab
G elongation factor mitochondrial 2 (*GFM2*)	RRF2M	Q969S9	TVTDFMAQ	Destab
heterogeneous nuclear ribonucleoprotein A3 (*HNRNPA3*)	ROA3	P51991	SREDSVKP	Stab
heat shock protein family D (Hsp60) member 1 (*HSPD1*)	CH60	P10809	VGYDAMAG	Stab
microtubule associated protein 1S (*MAP1S*)	MAP1S	Q66K74	DRVDAVLV	Stab
N(alpha)-acetyltransferase 15, NatA auxiliary subunit (*NAA15*)	NAA15	Q9BXJ9	HEADTANM	Stab
asparaginyl-tRNA synthetase (*NARS*)	SYNC	O43776	KKEDGTFY	Stab
3′-phosphoadenosine 5′-phosphosulfate synthase 1 (*PAPSS1*)	PAPS1	O43252	TGIDSEYE	Stab
phosphatase and actin regulator 2 (*PHACTR2*)	PHAR2	O75167	DSPDYDRR	Destab
POTE ankyrin domain family member F (*POTEF*)	POTEF	A5A3E0	ELPDGQVI	Stab
proteasome 26S subunit, ATPase 3 (*PSMC3*)	PRS6A	P17980	QEEDGANI	Stab
proteasome 26S subunit, ATPase 3 (*PSMC3*)	PRS6A	P17980	DILDPALL	Stab
glutamine rich 1 (*QRICH1*)	QRIC1	Q2TAL8	LTVDSAHL	Stab
RNA binding motif protein 22 (*RBM22*)	RBM22	Q9NW64	SNSDGTRP	Stab
RNA binding motif protein 39 (*RBM39*)	RBM39	Q14498	ERTDASSA	Stab
replication factor C subunit 1(*RFC1*)	RFC1	P35251	DEVDGMAG	Stab
splicing factor 3a subunit 1 (*SF3A1*)	SF3A1	Q15459	VTWDGHSG	Stab
signal recognition particle 54 (*SRP54*)	SRP54	P61011	QELDSTDG	Stab
transducin beta like 1X-linked (*TBL1X*)	TBL1X	O60907	TVFDGRPI	Stab
transducin beta like 1, Y-linked (*TBL1Y*)	TBL1Y	Q9BQ87	MEIDGDVE	Stab
tubulin alpha 3c (*TUBA3C*)	TBA3C	P0DPH7	VKCDPRHG	Stab
tubulin alpha 4a (*TUBA4A*)	TBA4A	P68366	IQPDGQMP	Stab
zinc finger CCCH-type containing 15 (*ZC3H15*)	ZC3HF	Q8WU90	VYIDARDE	Stab

^1^ stab: stabilizing after caspase cleavage, destab: destabilizing effect, according to the Arg/N-degron pathway.

## Supplementary Materials

The following are available online at Harvard Dataverse, https://doi.org/10.7910/DVN/OT9VSD, Figure S1: Distribution of P1 glutamate cleavage sites in three vertebrate classes, Figure S2: Principal coordinate analysis (PCoA) of cleavage sites and 60-amino acid sequences, Figure S3: Hierarchical clustering of cleavage sites, Figure S4: Hierarchical clustering of 60-amino acid sequences, Figure S5: Correlation between hydrophobicity of cleavage site surroundings and cleavage rates for human caspase targets, Figure S6: Distribution of P1′ destabilizing amino acid residues among cleavage sites and among vertebrates, Table S1: Human caspase targets, Table S2: Description of orthologous caspase targets, Table S3: Percentage of P1 glutamate cleavage sites in species, Table S4: Summary of conservation criteria, for all human and orthologous targets, Table S5: Secondary structure predictions for human caspase targets, Table S6: Comparison of secondary structure prediction results with previous studies, Table S7: Percentage of P1 prime destabilizing amino acids according to the Arg/N-degron pathway, at the species level, Table S8: Subset of caspase targets most important in the apoptotic program.

## Appendix A

**Table A1:** Clarification note for Table S1 ^1^.

Uniprot ID	Original Gene Names	Gene Name in Table S1
Q99666	*RGPD5, RGPD6*	*RGPD5*
Q13748	*TUBA3C, TUBA3D*	*TUBA3*
P62805	*HIST1H4A, HIST1H4B, HIST1H4C, HIST1H4D, HIST1H4E, HIST1H4F, HIST1H4H, HIST1H4I, HIST1H4J, HIST1H4K, HIST1H4L, HIST2H4A, HIST2H4B, HIST4H4*	*HIST*

^1^ There are 3 Uniprot IDs with excessive numbers of corresponding genes. We decided to leave one gene name for each of the Uniprot IDs.

**Table A2:** Column names and descriptions for Table S2.

Column Name	Description
h uniprot id	Human Uniprot ID
h protein symbol	Human gene symbol (Uniprot)
h gene symbol	Human protein symbol (Uniprot)
v gene name	Vertebrate gene name
v accession	NCBI accession number of the vertebrate protein sequence
class	Class
order	Order
family	Family
genus	Genus
species	Species
n matches with humans	Number of vertebrate caspase targets in a species’s proteome
total n proteins	Total number of proteins in a species’s proteome
h 60 aa	Human 60-amino acid sequence
v 60 aa	Vertebrate 60-amino acid sequence
v pblast evalue	pBLAST e-value for the vertebrate 60-amino acid sequences
v pblast score	pBLAST score for the vertebrate 60-amino acid sequences
v pblast identity	pBLAST identity for the vertebrate 60-amino acid sequences
h cleavage site	Human cleavage site
v cleavage site	Vertebrate cleavage site
v p1 aa	Vertebrate P1 amino acid
hamdist	Hamming distance estimate between human and vertebrate cleavage sites
h p1 pr	P1′ amino acid in the human cleavage site
h p1 pr effect	Effect of P1′ amino acid in the human cleavage site
v p1 pr	P1′ amino acid in the vertebrate cleavage site
v p1 pr effect	Effect of P1′ amino acid in the vertebrate cleavage site
v hydr index 20	Sum of hydrophobicity estimates for the central 20 amino acids in vertebrate 60-amino acid sequences
v hydr prev	Hydrophobicity prevalence values in vertebrate 60-amino acid sequences

### Supporting Information for Table S4: Summary of Conservation Criteria, for All Human and Orthologous Targets

Occurrence in seven classes shows the number of vertebrate classes having at least one orthologous caspase target. Occurrence in 328 species represents the number of vertebrate species from Table S2 (without humans) having at least one orthologous caspase target.

### Supporting Information for Table S5: Secondary Structure Predictions for Human Caspase Targets

Only sequences with a length of 60 amino acids were used in the analysis.

### Supporting Information for Table S6: Comparison of Secondary Structure Prediction Results with Previous Studies

Methods, databases, and tools used to predict secondary structure: PDB [65], modBase [66], PSIPRED [67], algorithm developed by Barkan et al. 2010 [68], MuFold [28].
